# Where Two Are Fighting, the Third Wins: Stronger Selection Facilitates Greater Polymorphism in Traits Conferring Competition-Dispersal Tradeoffs

**DOI:** 10.1371/journal.pone.0147970

**Published:** 2016-02-04

**Authors:** Adam Lampert, Tsvi Tlusty

**Affiliations:** 1 School of Human Evolution and Social Change, Arizona State University, Tempe, AZ 85287, United States of America; 2 Mathematical, Computational and Modeling Sciences Center, Arizona State University, Tempe, AZ 85287, United States of America; 3 Simons Center for Systems Biology and School of Natural Sciences, Institute for Advanced Study, Princeton, NJ 08540, United States of America; 4 Department of Physics and Center for Soft and Living Matter, Institute for Basic Science (IBS), UNIST, UNIST-gil 50, Ulsan, 689-798, South Korea; Chung-Ang University, REPUBLIC OF KOREA

## Abstract

A major conundrum in evolution is that, despite natural selection, polymorphism is still omnipresent in nature: Numerous species exhibit multiple morphs, namely several abundant values of an important trait. Polymorphism is particularly prevalent in asymmetric traits, which are beneficial to their carrier in disruptive competitive interference but at the same time bear disadvantages in other aspects, such as greater mortality or lower fecundity. Here we focus on asymmetric traits in which a better competitor disperses fewer offspring in the absence of competition. We report a general pattern in which polymorphic populations emerge when disruptive selection increases: The stronger the selection, the greater the number of morphs that evolve. This pattern is general and is insensitive to the form of the fitness function. The pattern is somewhat counterintuitive since directional selection is excepted to sharpen the trait distribution and thereby reduce its diversity (but note that similar patterns were suggested in studies that demonstrated increased biodiversity as local selection increases in ecological communities). We explain the underlying mechanism in which stronger selection drives the population towards more competitive values of the trait, which in turn reduces the population density, thereby enabling lesser competitors to stably persist with reduced need to directly compete. Thus, we believe that the pattern is more general and may apply to asymmetric traits more broadly. This robust pattern suggests a comparative, unified explanation to a variety of polymorphic traits in nature.

## 1 Introduction

Individuals in a population of a given species often exhibit very different trait values, owing either to genetic variability or to purely phenotypic plasticity even when their genes associated with the trait are identical. When the trait is continuous, such as a characteristic size or timing, the population may exhibit a unimodal trait distribution centered around a single abundant trait value, but may also exhibit a multimodal distribution with several characteristic trait values (morphs) [1–7]. Particularly, high degrees of polymorphism that encompasses traits with three or more morphs are widespread and were found in various species, including body-sizes of male fish [8], fertilization-timings in plants [2], dispersal-rates in insects [1], cooperation magnitudes in bacteria [9], seed sizes in plants [10], and horn sizes in male dung beetles [7].

It has been suggested that the underlying mechanism for polymorphism is negative density-dependence: Fitness may depend on the density of the morph in the population, which may promote coexistence between distinct morphs [3, 4, 11–13]. Specifically, in the context of resource competition, it has been suggested that such polymorphism may emerge via resource partitioning where several resource types are present, and each morph fits to a particular type of resource (character displacement, [14–16]). For example, if each bird has a beak size that fits a particular seed size, then selection may favor the beak size that fits the most abundant seed size, but selection may also favor several coexisting beak-sizes, each of which is specialized in consuming a particular seed size. In the latter case, the number of coexisting morphs (beak sizes) depends on the effective number of resources (seed sizes), which in turn depends on the ratio between the range of resource types and the range of resources that are being consumed effectively by a given consumer.

Polymorphism, however, may also occur when the competition is over a single resource. Competition is then asymmetric, namely, the trait provides some advantage while competing over the resource, but also bears some disadvantage such as greater mortality or less efficient dispersal [17–21]. Polymorphism in asymmetric traits is possible due to the ‘colonization-competition tardeoff’ [22, 23]. For example, seedlings initiated from larger seeds may have competitive advantage over those initiated from smaller seeds, but smaller seeds are cheaper to produce and therefore favorable in the absence of competition; this may lead to coexistence between small and large seeds [17]. For another example, hornless male beetles may sneak around and mate with females without encountering the stronger, horned males, which allows them to persist at a low abundance [24]. Nevertheless, the origin of highly polymorphic asymmetric traits with many morphs is still unclear. What is an intermediate seed-size [10] or horn-size [7] good for? Why does natural selection promote trimorphic horn sizes? The existence of generic mechanisms underlying polymorphism in asymmetric traits is therefore a basic question in understanding population structure and evolution.

In this paper, we focus on asymmetric traits in which the better competitor disperses fewer offspring in the absence of competition. We suggest a general explanation for polymorphism and we show that the number of stably-coexisting morphs increases with the strength of local, disruptive selection. Moreover, we use a variant of our model to show that the same mechanism may apply to cooperative traits that decrease the reproductive potential of their carrier but increases the reproductive potential of their neighbors. This idea is supported by previous studies [17, 21, 25, 26], each focused on a special case and demonstrates how the number of coexisting species increases with a certain environmental parameter that is equivalent to the strength of local disruptive selection. Hence, our study puts these observations in a general framework and suggests an underlying mechanism that may lead to the emergence of polymorphism.

## 2 Model With Local Selection and Global Dispersal

Populations in nature commonly are subdivided as each individual is spatially restricted and interacts locally with its neighbors for long periods of time, but eventually the individual migrates and then interacts with its new neighbors [27]. Dispersal may occur continuously over time, such as in infection diseases where bacteria or viruses migrate from host to host, or it may occur during a dispersal stage, such as with pollen of plants, with the larvae of benthic marine life, with the adolescents of many vertebrates and during the post-teneral migratory phase of adult insects [28, 29]. To examine polymorphism in asymmetric traits, we consider a population that is sub-divided into identical patches. Each individual is characterized by its ability to disperse its offspring to other patches, *q*, which may span over a continuous spectrum between high ability to disperse many offspring, *q* = 1, and low ability, *q* = 0. The trait is asymmetric as it bears some advantage in dispersal but also bears some disadvantage in local competition, namely, the lower the trait value *q*, the better the local (within-patch) competitive ability of its individual carrier.

**Fig 1:**
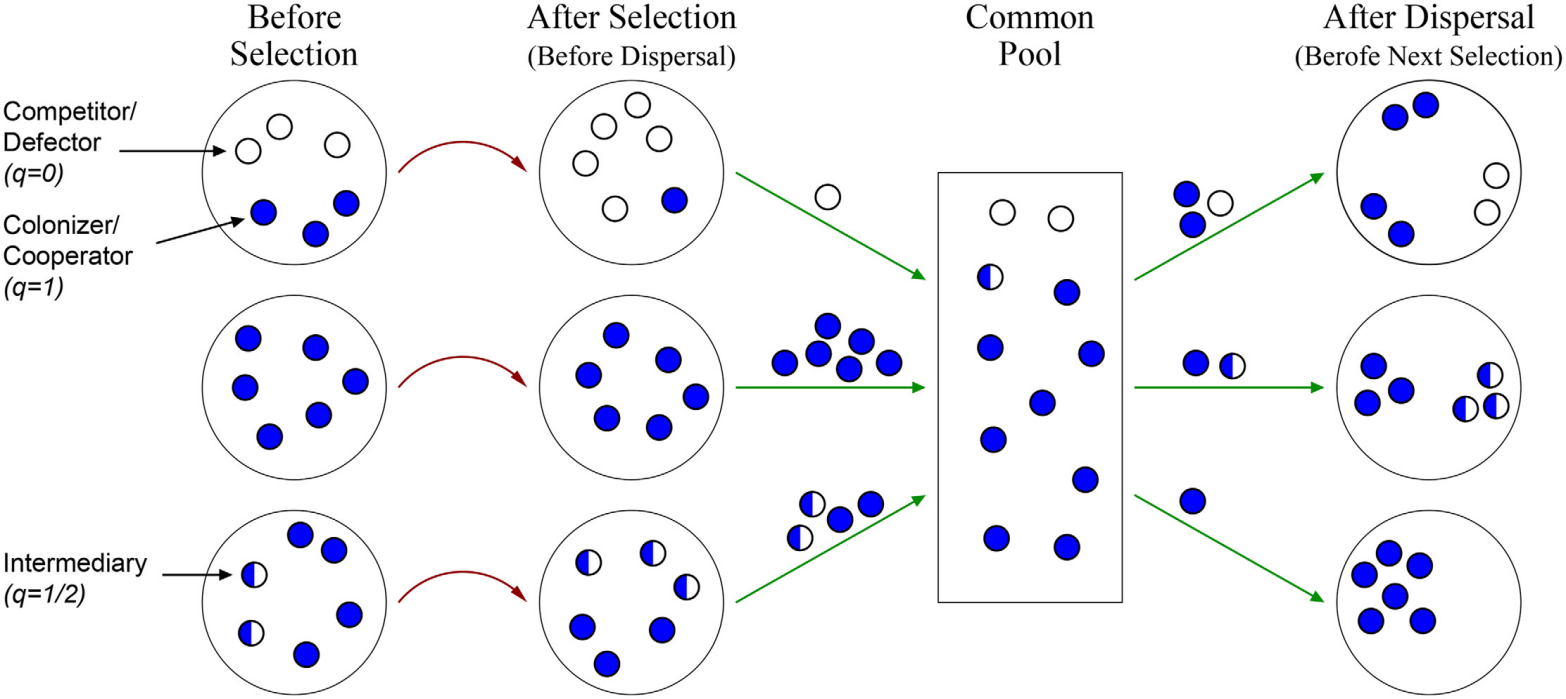
Our model comprises local selection and global dispersal. A single dynamical cycle is illustrated for 3 patches and 3 trait magnitudes (*q* = 0.1, *q* = 1/2, *q* = 1). At the selection stage (red curved arrows), deterministic selection that favors competitors with lower *q* takes place in each patch separately according to the replicator dynamics (Eq (1)), where the greater the disruptive selection strength, *s*, the greater the relative abundances of advantageous competitors become. At the dispersal stage (green straight arrows), the probability of each individual to join the common pool increases with the average *q* in its patch (or with its own *q* in an alternative model variation). Finally, each individual has a constant probability (∼*α*) to become a seeder and establish a clade in a randomly-picked patch. In our simulations, we consider a continuum of possible trait values, 0 ≤ *q* ≤ 1; large patch populations at selection stage; and infinitely many patches, which determines deterministic dynamics of trait abundances Eq (3).

Population dynamics comprise sequential cycles, each of which includes a within-patch selection stage followed by a dispersal stage (Fig 1) [17, 26, 29, 30]: Selection results in a greater success of better competitors (lower *q*), such as higher relative growth or higher chances to reproduce, whereas dispersal mixes the population and encompasses a certain advantage to lesser competitors (higher *q*). The selection stage takes place within each patch as the fraction of *q*-individuals in patch *j*, *n*_*q*, *j*_, evolves according to the replicator dynamics [31, 32]

$$\begin{array}{c} \frac{dn_{q,j}}{dt}=sn_{q,j}\left(F\left(q\right)-\Phi_{j}\right), \end{array}$$ (1)

where *s* > 0 is the selection strength, *F*(*q*) is the competitive fitness that monotonically decreases with *q*, and *Φ*_*j*_ is the average fitness in the patch. To determine the population at the end of a selection stage, Eq (1) is integrated over one unit of time, characterizing a single life cycle (e.g., typically, a single year). Note that, equivalently, time can be measured in any unit and the parameter *s* characterizes the selection strength times the duration of the stage.

Next, during the dispersal stage, populations of different patches are mixed as follows (Fig 1, see also [17, 26, 33, 34]). First, all individuals leave their prior patch and each individual arrives to a ‘common pool’ with a probability $\tilde{q}$; otherwise, the individual dies. We consider two variants of the model where the probability $\tilde{q}$ equals either Eq (1) *q* (asymmetric trait is modeled) or Eq (2) the average *q* in the patch (cooperation is modeled). Note that the common pool is an abstraction that is introduced for clarity (see also [17, 19, 26, 29]). Second, mutations that slightly tune *q* occur at a low rate *μ* (see below). Third, each individual in the pool has a given probability, *α*/*K*, to arrive at a randomly chosen patch; otherwise, the individual dies. Here *α* > 1 is a constant parameter and *K* ≫ 1 is the carrying capacity in each patch. Finally, at each patch, each seeder colonizes an equal fraction of the patch before the next selection stage begins. Note that the parameter *K* does not play a role in the dynamics; it is used simply to emphasize that the probability for becoming a seeder is low and only a few individuals are chosen to seed a patch. Therefore, although we assume that the population at selection stage is large and continuous (Eq (1)), the seeder population in each patch is finite (see also [17, 30]).

To avoid small population effects, we consider infinitely many patches. We consider the dynamics of *ρ*_*t*_(*q*), the distribution of trait *q* in the pool after *t* cycles [26]. This distribution is relative to the maximal occupancy, namely,

$$\begin{array}{c} \int_{0}^{1}\rho_{t}\left(q\right)dq=\hat{\Gamma }, \end{array}$$ (2)

where $\hat{\Gamma }\leq 1$ is the average fraction of individuals arriving to the common pool following selection, compared to the maximal possible arrival. In particular, $\hat{\Gamma }$ may be smaller than one either because some patches are not seeded during dispersal and are thus empty after selection, or because not all individuals arrive to the common pool if some have *q* < 1. Note that, although each patch is occupied by a finite number of traits, the overall distribution *ρ*_*t*_(*q*) may comprise a continuum of traits as we consider infinitely many patches, which does not limit the heterogeneity of the population in the common pool.

The description above well-defines our model dynamics and the dynamics of *ρ*_*t*_(*q*), but we describe here an equivalent representation for clarity. As we consider infinitely many patches, *ρ*_*t*_(*q*) follows deterministic dynamics. The idea is similar to Levins’ equation for metapopulations, dictating deterministic dynamics for the fraction of occupied patches in the limit of infinitely many stochastic patches [35] (and the same idea applies to cases where various patch occupancies are possible, including such with continuous traits [17, 21, 26, 30, 36]). To calculate *ρ*_*t*+1_(*q*) from *ρ*_*t*_(*q*), we express the dynamics as a sum over all possible patch configurations after dispersal. The distribution of traits at the common pool after *t* + 1 cycles, *ρ*_*t*+1_(*q*), follows

$$\begin{array}{ccc} \rho_{t+1}\left(q\right) & = & \frac{\rho_{t}\left(q\right)}{\hat{\Gamma }}\left[P_{1,t}q+P_{2,t}\int_{0}^{1}\rho_{t}\left(q^{\prime }\right)C_{2}\left(q;q^{\prime }\right)F\left(q;q^{\prime }\right)dq^{\prime }\right.\\ & + & \left.P_{3,t}\int_{0}^{1}\int_{0}^{1}\rho_{t}\left(q^{\prime \prime }\right)\rho_{t}\left(q^{\prime }\right)C_{3}\left(q;q^{\prime },q^{\prime \prime }\right)F\left(q;q^{\prime },q^{\prime \prime }\right)dq^{\prime }dq^{\prime \prime }+...\right]\;, \end{array}$$ (3)

where *P*_*n*, *t*_ is the probability that exactly *n* seeders colonize a given patch, *C*_*n*_(*q*; ⋅) is the fraction of *q*-individuals in a patch following selection stage (given the other *n* − 1 seeders that initiate the patch population), and *F*(*q*; ⋅) is the fraction of *q*-individuals that make it from the patch to the common pool after selection.

**Fig 2:**
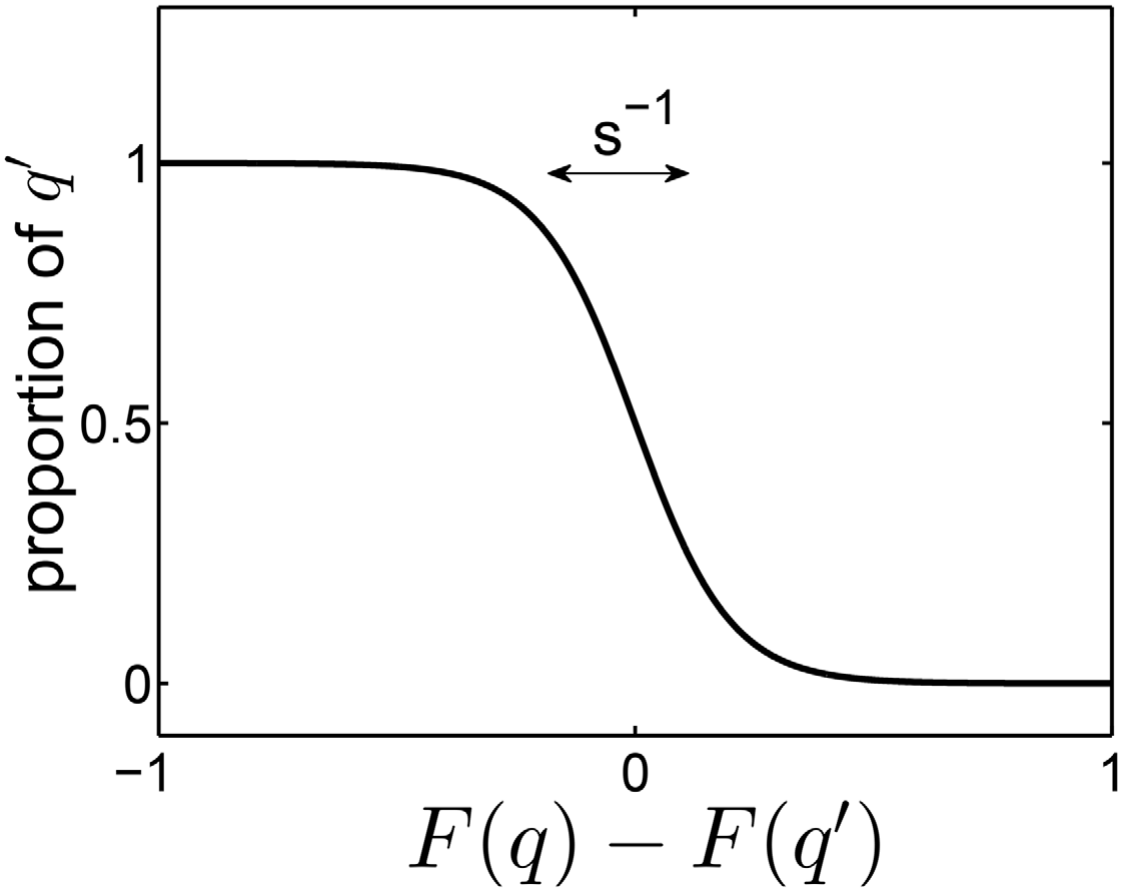
Selection imposes a characteristic length-scale along the *q*-axis. If a patch is seeded by two individuals with trait values *q* and *q*′, then, by the end of the selection stage, the fraction of *q*-individuals in the patch is given by *C*_2_(*q*′;*q*) = [*e*^*s*(*F*(*q*) − *F*(*q*′))^+1]^ − 1^, which is a sigmoidal curve with a characteristic length ∼1/*s*.

Since each individual may arrive at each patch with the same probability, *P*_*n*, *t*_ is given by a Poisson distribution, where the average number of seeders that arrive to each patch (according to the way we describe the model) the mean number of seeders is given by $\alpha \hat{\Gamma }$:

$$\begin{array}{c} P_{n,t}=\frac{{\left(\alpha \hat{\Gamma }\right)}^{n}e^{-\alpha \hat{\Gamma }}}{n!} \end{array}$$ (4)

(note that *P*_*n*, *t*_ depends on *t* as $\hat{\Gamma }$ depends on *t*). *C*_*n*_ is calculated from the replicator dynamics. Specifically, *C*_2_(*q*;*q*′) is the fraction of *q*-individuals in a patch after one unit of time following Eq (1), *n*_*q*, *j*_(*t* = 1), where initial conditions at *t* = 0 are *n*_*q*, *j*_ = 1/2 and *n*_*q*′, *j*_ = 1/2 (Fig 2); *C*_3_(*q*;*q*′, *q*″) is the same quantity, *n*_*q*, *j*_(*t* = 1), but with initial conditions *n*_*q*, *j*_ = 1/3, *n*_*q*′, *j*_ = 1/3, *n*_*q*″, *j*_ = 1/3; etc. *F*(*q*; ⋅) equals either *q* or the average *q* in the population after selection, depending on the variant. For example, if an asymmetric trait is considered, then *F*(*q*;*q*′, *q*″) = *q*, and if cooperation is considered, then *F*(*q*;*q*′, *q*″) = *C*_3_(*q*;*q*′, *q*″)*q*+*C*_3_(*q*′;*q*, *q*″)*q*′+*C*_3_(*q*″;*q*, *q*′)*q*″.

**Fig 3:**
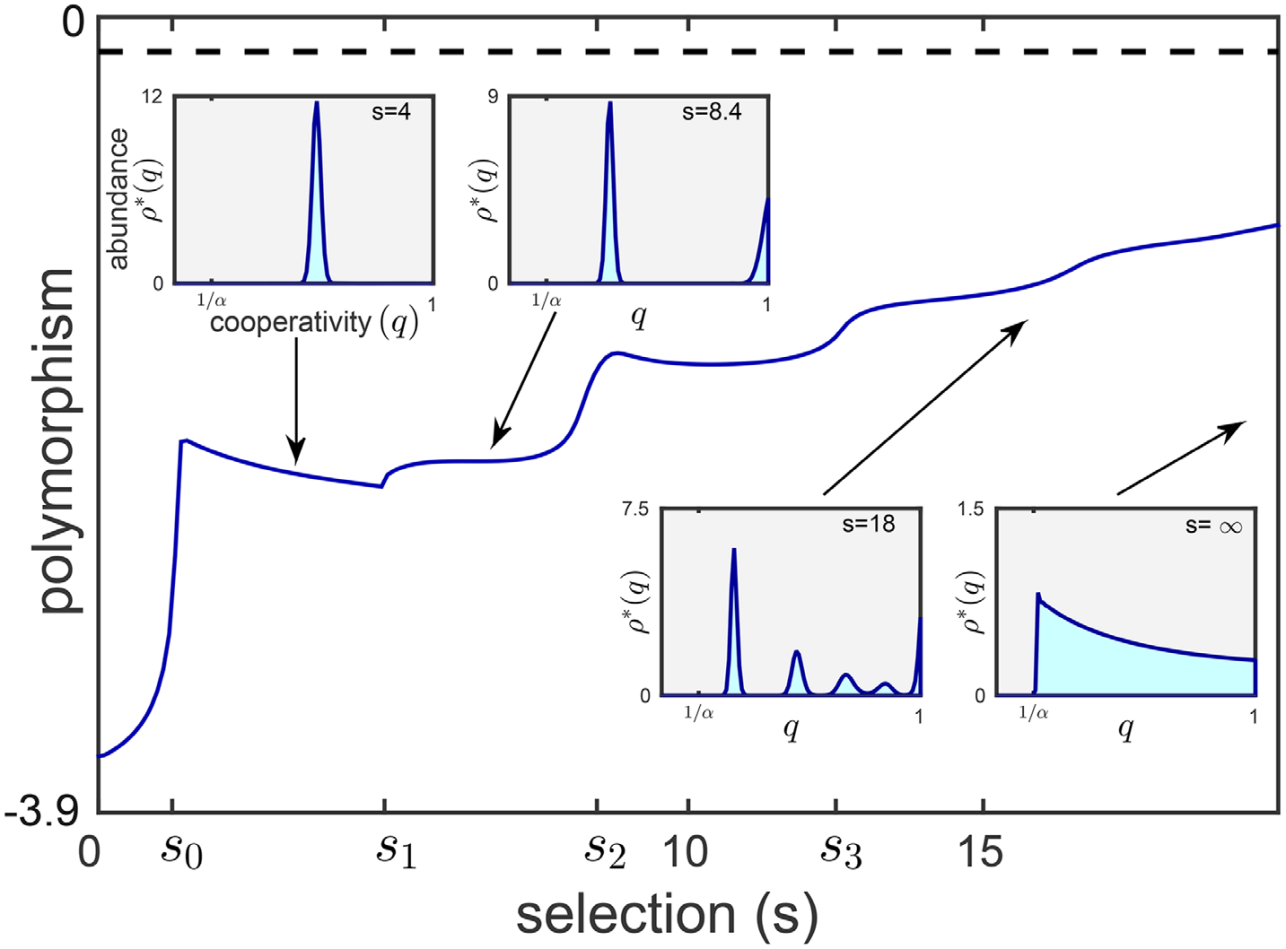
From monomorphic to polymorphic populations. When local selection is weak (*s* < *s*_1_), a single trait magnitude dominates at steady state (left inset). For stronger local competition, when *s* surpasses *s*_1_, two morphs with distinct trait magnitudes coexist (second inset). For even larger *s*, the steady state population becomes more and more polymorphic as more trait values coexist (third inset), until the entire spectrum 1/*α* < *q* ≤ 1 is stably populated when *s* is very large (right inset). The width of the packs corresponds to the mutation load, where no mutations implies sharp, zero-width peaks. The increased number of coexisting morphs, which appear at each *s*_*i*_, implies greater steady state polymorphism (main plot), which we measure as the distribution’s entropy (given by $H(\rho (q))=-\int_{0}^{1}\rho (q)\text{ln}(\rho (q))dq$, where a maximal possible value *H* = 0 that is exhibited when *ρ*(*q*) = 1). The polymorphism in our model asymptotically approaches *H*_∞_ = −ln^2^ *α*/(2*α*) (dashed line) as *s* → ∞. Parameters: *α* = 2.5, $\hat{\alpha }=3$, *μ* = 4, *F*(*q*) = −*q*, *y*-axis ∈[−3.9, 0], Δ*q* = 0.01.

**Fig 4:**
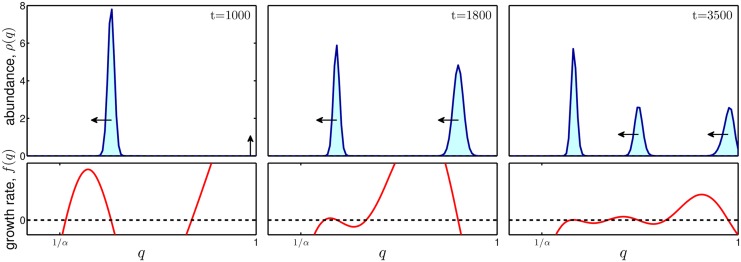
Emergence of polymorphic populations. Time evolution of the distribution of trait values, *ρ*_*t*_(*q*), (Eq (3)) is characterized by a series of emerging morphs with high values of *q*, which in turn evolve towards lower values of *q*. Morphs emerge when the densities of more competitive morphs become sufficiently low, which results in a positive per-capita, per-cycle growth, *f*(*q*), for sufficiently large *q*. The morphs evolve leftwards to greater competitiveness as long as mutants with slightly lower *q* have a positive *f*(*q*). Here the cooperative trait variant of our model is considered. Parameters: *s* = 18, *α* = 2.5, $\hat{\alpha }=3$, *μ* = 1, *F*(*q*) = −*q*, Δ*q* = 0.01.

**Fig 5:**
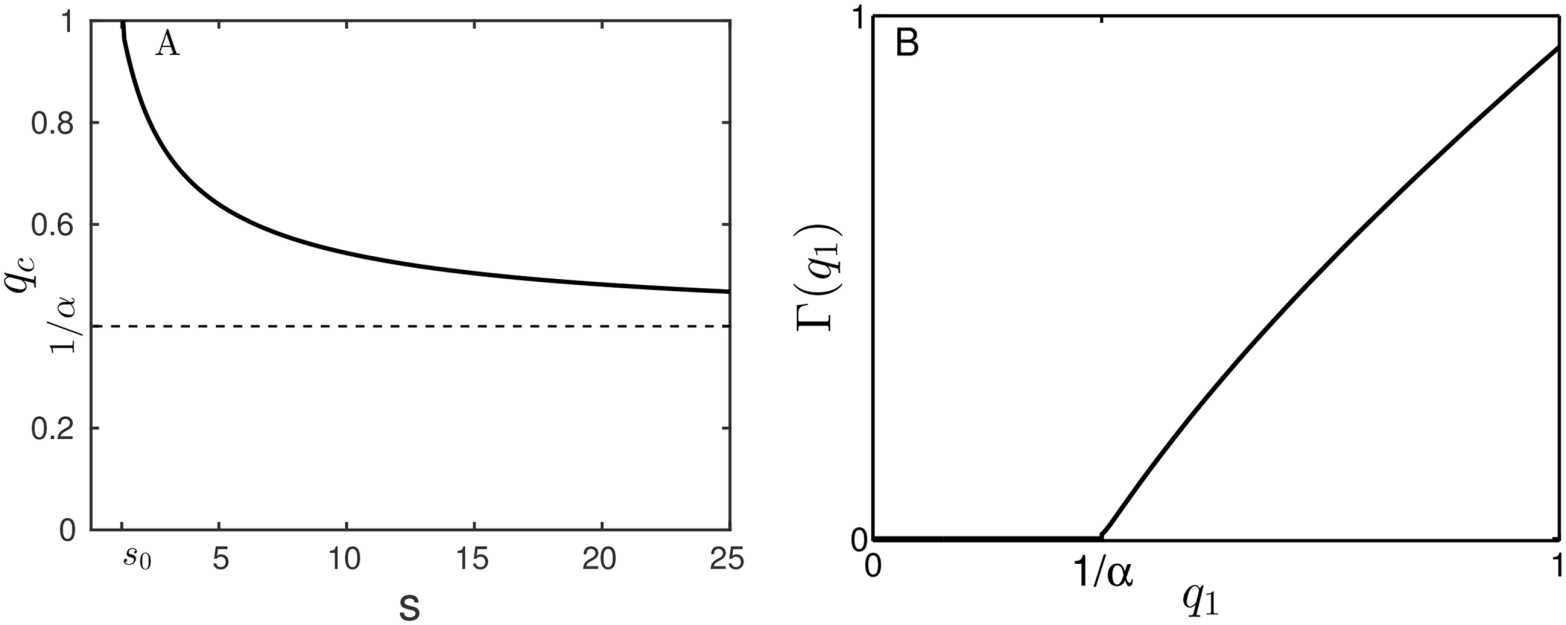
Steady-state of a monomorphic population with trait value *q*_1_. (A) Over time, mutations drive *q*_1_ towards 1 if *s* < *s*_0_ and towards *q*_*c*_ otherwise (*q*_*c*_ is the convergence stable strategy [38, 52]). (B) The steady state total abundance of the population approaches Γ(*q*_1_) at steady state: $\int_{0}^{1}\rho (q)dq=\int_{0}^{1}\Gamma (q_{1})\delta (q-q_{1})dq=\Gamma (q_{1})$.

In our numerical simulations (Figs 3 and 4), we considered a dense grid with a resolution Δ*q* along the space of trait values *q*. For computational tractability, we assumed that the number of seeders in a patch is bounded from above by $\hat{\alpha }$, and we verified via simulations with lower resolution along the *q*-axis that our results are qualitatively valid without this bound (in our analytic analysis, as well as in Fig 5 and S1 Fig, we do not use this bound). Next, for computational-efficiency, we calculated in advance the time-independent functions *C*_*n*_ for all initial species configurations. Then, to simulate the dynamics of *ρ*_*t*_(*q*), we substituted the corresponding values (see also [26]). Finally, we simulate mutations as a time discrete diffusion process with coefficient *μ* along the *q*-axis [21, 26]: Each cycle, for each point *q*_0_ on the grid, we added *μ* × (Δ*q*)^2^ × [*ρ*_*t*_(*q*_0_ − Δ*q*) − 2*ρ*_*t*_(*q*_0_) + *ρ*_*t*_(*q*_0_ + Δ*q*)], and we considered reflecting boundary conditions, *ρ*_*t*_(−Δ*q*) = *ρ*_*t*_(0) and *ρ*_*t*_(1 + Δ*q*) = *ρ*_*t*_(1).

## 3 Stronger Selection Result in Greater Polymorphism

### 3.1 General pattern: numeric analysis

We start with a general description of our simulation results and analysis of the model (Figs 3 and 4). The following pattern characterizes both variants, asymmetric trait and cooperation (although Figs 3 and 4 demonstrate the cooperation variant, the main conclusions are the same). Our results show that polymorphism increases with selection strength. When selection, *s*, is weak, population composition in each patch hardly changes during selection stage, and efficient dispersers (*q* = 1) are advantageous over competitors. But as *s* surpasses a certain threshold, *s*_0_, individuals with *q* slightly smaller than 1 become advantageous. Consequently, evolution drives the population towards lower values of *q* until it reaches a certain evolutionarily stable value, *q*_*c*_(*s*), which is the single dominant morph (Fig 3, left inset).

As selection further increases, competition becomes more significant, *q*_*c*_(*s*) further decreases (Fig 5A) and, consequently, the overall population abundance decreases (Fig 5B). Furthermore, as *s* surpasses another threshold, *s*_1_, individuals with *q* = 1 invade and stably coexist with the subpopulation at *q*_*c*_(*s*), owing to the increased fraction of unoccupied patches (Fig 5B). This may give rise to a dimorphic populations with two morphs, one at *q* = *q*_*c*_(*s*) and one at *q* = 1 (Fig 3, second inset).

Next, as *s* further increases, the emerging morph at *q* = 1 also undergoes the same process and moves leftwards to lower values of *q*. For even stronger selection, evolution comprises sequential events of emerging new morphs of highly cooperative individuals, which in turn gradually move to lower cooperation magnitudes (Fig 4). The resulting steady state population becomes more and more polymorphic with an increasing number of coexisting morphs of different cooperation magnitudes *q* (Fig 3, third inset). Finally, extreme competitive selection may promote smooth steady state distribution with even greater polymorphism (Fig 3, right inset).

### 3.2 Time evolution: adaptive dynamics analysis

#### 3.2.1 General case

Here, we derive some analytic results for our model using adaptive dynamics analysis [37, 38]. Assume that, initially, all individuals have the same trait value, *q*_1_. Also assume that fixation rate is much higher than mutation rate, and hence, *q*_1_ slowly evolves, while the abundance of the *q*_1_-population in the pool, Γ(*q*_1_), instantaneously adapts to the current value of *q*_1_, namely, *ρ*(*q*)≈Γ(*q*_1_)*δ*(*q* − *q*_1_) where *δ* is a delta function. Since the number of seeders initiating patch populations is given by a Poisson distribution with mean *α*Γ(*q*_1_), it follows that the fraction of empty patches is given by exp(−*α*Γ(*q*_1_)). Moreover, if *q*_1_ < 1/*α*, the population is unviable (Γ(*q*_1_) = 0) as each populated patch contributes less than one seeder to the next cycle. Therefore,

$$\begin{array}{c} \Gamma \left(q_{1}\right)=\left\{\begin{array}{ccc} 0 & \text{if} & 0\leq q_{1}\leq 1/\alpha \\ q_{1}\left(1-e^{-\alpha \Gamma (q_{1})}\right) & \text{if} & 1/\alpha <q_{1}\leq 1 \end{array}\right. \end{array}$$ (5)

(Fig 5B; see also [17]). Note that the Eq (5) is valid for both variants as they are indistinguishable where only one type exists. Eq (5) can also be derived directly from Eq (3) as the solution where *ρ*_*t*+1_(*q*_1_) = *ρ*_*t*_(*q*_1_), noting that *ρ*_*t*_(*q*) = Γ(*q*_1_)*δ*(*q* − *q*_1_), $\Gamma (q_{1})=\hat{\Gamma }$, *C*_*n*_(*q*_1_;*q*_1_, …) = 1 and *F*(*q*_1_;*q*_1_, …) = *q*_1_.

Next, mutants with *q*′ ≈ *q*_1_ invades the resident *q*_1_-population if and only if a *q*′-seeder produces, on average, more than one seeder for the subsequent dispersal, provided that *q*′-seeders are scarce and no more than one *q*′ individual seeds the same patch [17, 19, 30]. If a *q*′-individual seeds a patch that is also seeded by exactly *M* *q*_1_-seeders, then it follows from Eq (1) that its proportion in the patch by the end of the selection stage is given by

$$\begin{array}{c} n_{q^{\prime }}=\frac{e^{s\left(F\left(q^{\prime }\right)-F\left(q_{1}\right)\right)}}{M+e^{s\left(F\left(q^{\prime }\right)-F\left(q_{1}\right)\right)}}\;, \end{array}$$ (6)

which is a sigmoidal curve with a characteristic length ∼1/*s* along the *q*-axis (Fig 2). Therefore, in the asymmetric trait variant, the *q*′-seeder contributes on average

$$\begin{array}{c} f_{M}\left(q^{\prime };q_{1}\right)=\frac{e^{s\left(F\left(q^{\prime }\right)-F\left(q_{1}\right)\right)}}{M+e^{s\left(F\left(q^{\prime }\right)-F\left(q_{1}\right)\right)}}\times \alpha q \end{array}$$ (7)

while in the cooperation variant, the *q*′-seeder contributes on average

$$\begin{array}{c} f_{M}\left(q^{\prime };q_{1}\right)=\frac{e^{s\left(F\left(q^{\prime }\right)-F\left(q_{1}\right)\right)}}{M+e^{s\left(F\left(q^{\prime }\right)-F\left(q_{1}\right)\right)}}\times \alpha \frac{e^{s\left(F\left(q^{\prime }\right)-F\left(q_{1}\right)\right)}q^{\prime }+Mq_{1}}{M+e^{s\left(F\left(q^{\prime }\right)-F\left(q_{1}\right)\right)}} \end{array}$$ (8)

*q*′-seeders to the next cycle. The per-capita, per-cycle growth in the *q*′-population, *f*(*q*′;*q*_1_), is given by the average of *f*_*M*_ over *M*, weighted by the probability of having exactly *M* seeders in a patch:

$$\begin{array}{c} f\left(q^{\prime };q_{1}\right)=\sum_{M=0}^{\infty }P_{M}\left(q_{1}\right)f_{M}\left(q^{\prime },q_{1}\right)-1\;, \end{array}$$ (9)

where

$$\begin{array}{c} P_{M}\left(q_{1}\right)={\left(\alpha \Gamma (q_{1})\right)}^{M}e^{-\alpha \Gamma (q_{1})}/M! \end{array}$$ (10)

is the probability that a patch is seeded by exactly *M* *q*_1_-individuals (Poisson distribution). The term −1 is present in Eq (9) because *f*(*q*′;*q*_1_) characterizes the growth rate, which equals zero if each seeder produces, on average, exactly one seeder for the next generation.

For all *q*_1_, *f*(*q*_1_;*q*_1_) = 0 and a *q*′ mutant invades if and only if *f*(*q*′;*q*_1_) > 0. Therefore, the evolutionary dynamics of *q*_1_ follows the selection gradient *D*(*q*_1_) = (*df*(*q*′, *q*_1_)/*dq*′)|_*q*′ = *q*_1__[19, 38]. Assuming the asymmetric trait variant, this implies

$$\begin{array}{c} D\left(q_{1}\right)=\frac{df(q^{\prime },q_{1})}{dq^{\prime }}{|}_{q^{\prime }=q_{1}}=\alpha \sum_{M=0}^{\infty }P_{M}\left(q_{1}\right)\frac{1+MsF^{\prime }\left(q_{1}\right)}{{\left(M+1\right)}^{2}}\;. \end{array}$$ (11)

The evolution of a *q*_1_-population stops either where *q*_1_ reaches a bound (*q*_1_ = 1) and cannot further increase, or where *q*_1_ approaches *q*_*c*_ that zeroes the selection gradient (*D*(*q*_*c*_) = 0), which implies

$$\begin{array}{c} \sum_{M=0}^{\infty }\frac{{\left(\alpha \Gamma \left(q_{c}\right)\right)}^{M}e^{-\alpha \Gamma (q_{c})}\left(1+MsF^{\prime }\left(q_{c}\right)\right)}{{\left(M+1\right)}^{2}M!}=0. \end{array}$$ (12)

#### 3.2.2 Strong selection implies small distances between morphs

To solve Eq (12), we assume *s* ≫ 1, which implies Γ(*q*_*c*_) ≪ 1/*α*. The major contribution to the sum originates from the terms with *M* = 0 and *M* = 1, which implies

$$\begin{array}{c} 0=1+\alpha \Gamma \left(q_{c}\right)\frac{1+sF^{\prime }\left(q_{c}\right)}{4}+O\left(s^{-2}\right). \end{array}$$ (13)

Note that, in this limit, the two variants of the model, asymmetric trait and cooperation, become identical, because each patch is either empty or is occupied by identical individuals with same value of *q*. For Γ(*q*_*c*_) ≪ 1/*α*, *e*^−*α*Γ(*q*_*c*_)^ ≈ 1 − *α*Γ(*q*_*c*_), and substitution into Eq (5) yields Γ(*q*_*c*_) ≈ 2(*q*_*c*_ − 1/*α*). Substitution into Eq (13) yields

$$\begin{array}{ccc} & & q_{c}=\frac{1}{\alpha }+\frac{2}{sF^{\prime }\left(1/\alpha \right)}+O\left(s^{-2}\right),\\ & & \Gamma \left(q_{c}\right)=\frac{4}{sF^{\prime }\left(1/\alpha \right)}+O\left(s^{-2}\right). \end{array}$$ (14)

Next, we analyze the dynamics of the next branch at *q* = *q*_2_. Since initially *q*_2_ > *q*_*c*_ and *s* ≫ 1, we assume that only patches that are seeded solely by *q*_2_-individuals (without *q*_1_-individuals) contribute *q*_2_-individuals to the common pool following selection. Equivalently, the *q*_2_-population may populate only the fraction 1 − Γ(*q*_*c*_) from the patches, and therefore, for any viable *q*_2_, it follows that Γ_2_(*q*_*c*_2__) = 2(*q*_*c*_2__ − 1/*α* − 4/*s*)+*O*(*s*^−2^), which implies

$$\begin{array}{ccc} & & q_{c_{2}}=\frac{1}{\alpha }+\frac{6}{sF^{\prime }\left(1/\alpha \right)}+O\left(s^{-2}\right),\\ & & \Gamma_{2}\left(q_{c_{2}}\right)=\frac{4}{sF^{\prime }\left(1/\alpha \right)}+O\left(s^{-2}\right). \end{array}$$ (15)

#### 3.2.3 Extreme selection implies smooth distribution

When selection is extremely strong (*s* = ∞), a smooth distribution of cooperation magnitudes emerges, which is consistent with previous studies [21, 39, 40]. When *s* = ∞, each patch is occupied by a single species by the end of each selection stage. Therefore, without mutations,

$$\begin{array}{c} \rho_{t+1}\left(q\right)=qd_{t+1}\left(q\right), \end{array}$$ (16)

where *d*(*q*) is the fraction of patches occupied by *q*-investors by the end of selection stage. Without mutations, *d*_*t*+1_(*q*) is proportional to the number of *q*-seeders, times the probability that no seeder with *q*′ < *q* arrives at the patch, *P*(*q* = *q*_*min*_), and therefore,

$$\begin{array}{c} d_{t+1}\left(q\right)=\alpha \rho_{t}\left(q\right)P\left(q=q_{min}\right). \end{array}$$ (17)

Substitution into Eq (16) yields

$$\begin{array}{c} \rho_{t+1}\left(q\right)=\rho_{t}\left(q\right)\left[\alpha qP(q=q_{min})\right]. \end{array}$$ (18)

The distribution of competitor seeders with *q*′ < *q* is Poissonian with a mean $\lambda_{q}=\alpha \int_{0}^{q}\rho_{t}(q^{\prime })dq^{\prime }$, and therefore, $P(q=q_{min})=e^{-\lambda_{q}}$, which implies

$$\begin{array}{c} \rho_{t+1}\left(q\right)=\rho_{t}\left(q\right)\left[\alpha qe^{-\alpha \int_{0}^{q}\rho_{t}\left(q^{\prime }\right)dq^{\prime }}\right]. \end{array}$$ (19)

The steady state distribution of cooperation magnitudes, ρ*(*q*), solves *ρ*_*t*+1_(*q*) = *ρ*_*t*_(*q*): for all *q*, either ρ*(*q*) = 0 or

$$\begin{array}{c} \alpha qe^{-\alpha \int_{0}^{q}\rho^{*}\left(q^{\prime }\right)dq^{\prime }}=1. \end{array}$$ (20)

If *q* < 1/*α*, then ρ*(*q*) = 0 is the only solution, while if *q* > 1/*α*, then a solution for Eq (20) exists. Eventually, the dynamics implies the steady state

$$\begin{array}{c} \rho^{*}\left(q\right)=\left\{\begin{array}{ccc} 0 & \text{if} & 0\leq q\leq 1/\alpha \\ {\left(\alpha q\right)}^{-1} & \text{if} & 1/\alpha \leq q\leq 1, \end{array}\right. \end{array}$$ (21)

and the total steady state species abundance is given by

$$\begin{array}{c} \int_{0}^{1}\rho^{*}\left(q\right)dq=\frac{ln(\alpha )}{\alpha }\;. \end{array}$$ (22)

Note that, mathematically, the reason why the continuous distribution is possible is that the sigmoidal function in Fig 2 is no longer a smooth function as it becomes a step function where *s* → ∞ [40]. Such discontinuities are known to create the artifact of infinitely close phenotype packing, but are also known not to be biologically realistic [41].

## 4 Discussion

We studied the evolution of continuous asymmetric traits that entail a tradeoff between better ability to compete locally with neighboring population and better ability to disperse offspring. Specifically, in one variant we considered a tradeoff between local competition and global dispersal (colonization-competition tradeoff, [17, 21–23, 42]); in another variant we considered a cooperative trait that increases the ability of neighbors to disperse offspring but incurs an individual cost [26, 29, 43–45]. In both variants, we considered two basic parameters: strength of local selection, *s*, and potential mean number of dispersers that initiate a population in a newly formed patch (seeders), *α*. We demonstrated the following general pattern: The steady state distribution of traits values comprises (1) a single dominant trait value when selection is weak, (2) increasing number of dominant values with increased selection, and (3) a continuous spectrum of coexisting trait values at extreme selection levels. In line with our theory, populations in nature range from monomorphic to highly polymorphic ones [1, 9, 10].

The pattern of increased polymorphism with increased selection appears in both model variants, because the underlying mechanism is general and apply to both variants. The underlying mechanism, in which increased selection results in lower abundance, demonstrates the phrase “when two are fighting, the third wins”: Two individuals (or lineages) competing for a resource may lose the battle to a third individual that abandoned fighting and is looking instead for an alternative, unoccupied resource patch. More generally, if several individuals that did not fight find their way to another resource patch, these individuals will eventually have to compete among themselves. This ultimately leads to the evolution of hierarchical competitive abilities.

It is well-known that the interplay between local selection and global migration may promote coexistence between cooperators and defectors, owing to the ability of groups of cooperators to populate empty patches [29, 44]. Similarly, in the context of ecological communities, it was suggested that species with higher fecundity may stably sustain by colonizing patches that are unoccupied by species with stronger competitive abilities (colonization-competition tradeoff), which may promote coexistence between several species with different trait values [22, 23]. Moreover, it was shown that moderated strengths of local disruptive selection may promote multimodal body-size distributions [21], and may also promote coexistence between a few branches of coexisting species at the community level [17, 18, 25, 42]. In the present study, we demonstrated that the increment in polymorphism with increased selection is a consequence of the evolutionary dynamics and is insensitive to the form of the fitness function. Stronger selection amplifies the evolution towards higher competitiveness, which in turn leads to lower density and gives rise again to lesser competitors. Therefore, we suggest that the same general evolutionary dynamics of asymmetric traits may be a basic mechanism that governs populations at both single species and community level.

We emphasize that we adopted the well-established adaptive dynamics approach, focusing on the long-term evolutionary dynamics at the phenotypic level and neglecting epistasis [38, 46]. Moreover, we considered identical patches in a constant environment. Thereby, we could focus on the emergence of polymorphism due to the tradeoff between selection and dispersibility. This is fundamentally different from polymorphism caused by the presence of various patch types (either in time or in space) [47–49]. Specifically, the fitness in our model is defined such that the same phenotype is always favorable during within-patch selection. Note that our results are general and are insensitive to the fitness function, *F*(*q*). Different choices of the fitness function may alter the regions along the *q*-axis where polymorphism occurs, but the general trend remains unchanged for any smooth fitness function. Specifically, our results show that, when selection is sufficiently strong, the first few morphs approach a distance of about 4/(*sF*′(1/*α*)) from one another (Eqs (14) and (15)). This demonstrates that the distance between morphs, as well as their abundance, decreases as selection increases. The pattern is also independent of the potential mean number of seeders, *α*, as the critical threshold for transition from monomorphic to dimorphic population, *s*_1_ (Fig 3), does not diminish with *α* (S1 Appendix and S1 Fig).

Our results suggest a new perspective on the evolutionary dynamics of cooperative traits that unites current paradigms. May and Nowak (1994) [50] studied virulence, in which more virulent individuals take over the within-host population, but at an increased risk of their host death, thereby harming the within-host population as a whole (defectors). Their model suggests a continuous spectrum of virulence levels at steady state [39, 50]. In contrast, Ackerman *et al*. (2008) [30] studied viral lysis, in which viruses are self-sacrificed to enable the invasion of their allies into hosts (cooperators), where each virus is characterized by its sacrifice probability (cooperation magnitude). Their model exhibits a steady state with a single, dominant sacrifice probability. Similarly, a general model by Doebeli *et al*. (2004) [19] also demonstrated populations with one or two cooperation levels at steady state. Although all the above models assume basically the same mechanism of group selection, in which within-group (host) increase of defectors is compensated by the greater success of cooperator-rich groups, they demonstrate very different evolutionary dynamics of trait distributions. In this study we showed that the seemingly contradicting results are instances of a more general pattern, in which more cooperation levels coexist as within-patch selection increases: When local selection is relatively strong, and within-patch population saturates before individuals are dispersed to other patches (as in [50]), the steady state distribution is continuous. At the other extreme, when selection is relatively weak and the population composition hardly changes between consecutive dispersals (as in [19, 30]), there is a single dominant cooperation level. In between, a gradual increase in the number of coexisting cooperation levels is exhibited.

Finally, we formulated our model as competition over habitable patches, while in many cases the competition is over females, which may play a similar role in the dynamics. The density of more competitive males decreases since they mature later and are subject to greater predation risk, which results in a fewer competitive adults [8]. Indeed, many species exhibit dimorphism and trimorphism in male traits relating to competition over females. Examples include trimorphic male body-sizes in fish [8] and ispoods [51], and the size of some organs that serve as ‘weapons’ while combating other males in arthropods, such as horn-size in dung beetles, mandibles in lucanid beetles, and ventral spines in weevils [7]. The number of morphs may vary between one and three, depending on the particular subspecies [7]. This suggests that another factor, perhaps the ratio between disruptive selection strength and predation risk, affects the evolution of polymorphism in those species. While this idea requires further investigation, we believe that the mechanism underlying polymorphism that we presented here is general and may apply to a wide range of asymmetric traits.

## Supporting Information

S1 Appendix (PDF)

S1 Fig (PDF)
