# Supplementary material for: Where Two Are Fighting, the Third Wins: Stronger Selection Facilitates Greater Polymorphism in Traits Conferring Competition-Dispersal Tradeoffs
Source: PLoS One. 2016 Feb 4;11(2):e0147970. doi: 10.1371/journal.pone.0147970 (PMC4742239; doi:10.1371/journal.pone.0147970)
Supplement: S1 Appendix — (PDF) [file pone.0147970.s001.pdf]

Supporting Information

for the paper:

Where two are fighting, the third wins: Stronger selection facilitates greater polymorphism in traits conferring competition-dispersal tradeoffs

Adam Lampert and Tsvi Tlusty

S1 Appendix: Calculating the thresholds

Here, we calculate (i) s_0, the critical value of s above which $q = 1$ is not dominant, and (ii) s_1, the critical value of s above which the steady state population in our model becomes dimorphic (Fig. 3). To calculate s_0, note that if $s = s_0$, $D(1) = 0$. Therefore, it follows from Eq. (10) that

$$\sum_{M=0}^{\infty} P_M(1) \frac{1 - Ms_0F'(1)}{(M+1)^2} = 0 \; .$$

Since $Q_{-1}(\lambda) \equiv \sum_{M=0}^{\infty} P_M/(M+1) = \frac{1}{\lambda}(1-e^{-\lambda})$, it follows from Eq. (5) that $Q_{-1}(\alpha\Gamma(q_1)) = 1/(\alpha q_1)$, which implies

$$s_0 = \frac{1}{F'(1)} \frac{\alpha Q_{-2}(\alpha\Gamma(1))}{1 - \alpha Q_{-2}(\alpha\Gamma(1))} \; , \tag{S1}$$

where

$$Q_{-2}(\lambda) \equiv \sum_{M=0}^{\infty} P_M/(M+1)^2. \tag{S2}$$

Fig. S1 demonstrates that s_0 decreases with α, approaches infinity as $\alpha \to 1$, and approaches zero as $\alpha \to \infty$.

At the second threshold, where $s = s_1$, a mutant at $q = 1$ has an equal per-capita growth-rate as a q_c individual, namely $f(1, q_c) = 0$. In addition, since $D(q_c) = 0$, it follows that, for $F(q) = -q$,

$$s_1 = \frac{\alpha Q_{-2}(\alpha\Gamma(q_c))}{1 - \alpha q_c Q_{-2}(\alpha\Gamma(q_c))} \; . \tag{S3}$$

Finding q_c is more complicated, but we calculate a higher bound for s, $\hat{s}_1$, by assuming that the branch at $q = 1$ invades if and only if it can sustain by using empty patches that are not seeded by any q_c-seeder. Then, $f(1, q_c) = 0$ implies $\alpha P_0(q_c) = 1$, which yield $\exp(-\alpha\Gamma(q_c)) = 1/\alpha$. Thus,

$$\Gamma(q_c) = \frac{\ln(\alpha)}{\alpha}, \tag{S4}$$

and from Eq. (5) it follows that

$$q_c = \frac{\ln(\alpha)}{\alpha - 1}, \tag{S5}$$

and therefore,

$$\hat{s}_1 = \frac{\alpha Q_{-2}(\ln(\alpha))}{1 - \frac{\alpha \ln(\alpha)}{\alpha - 1} Q_{-2}(\ln(\alpha))}. \tag{S6}$$

Fig. S1 demonstrates that $\hat{s}_1$ approaches s_1 when α is large, and also that both s_1 and $\hat{s}_1$ approaches infinity either as $\alpha = 1$ or as $\alpha \to \infty$.
