# Supplementary material for: Where Two Are Fighting, the Third Wins: Stronger Selection Facilitates Greater Polymorphism in Traits Conferring Competition-Dispersal Tradeoffs
Source: PLoS One. 2016 Feb 4;11(2):e0147970. doi: 10.1371/journal.pone.0147970 (PMC4742239; doi:10.1371/journal.pone.0147970)
Supplement: S1 Fig — (PDF) [file pone.0147970.s002.pdf]

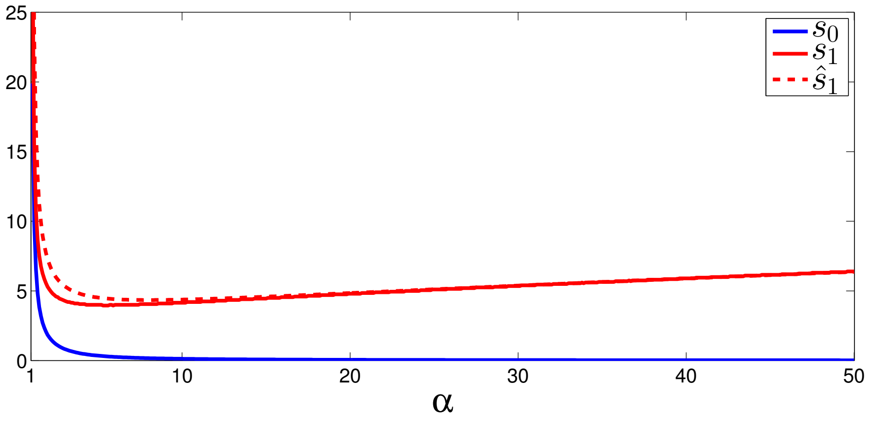

S1 Figure: *Robust transition from monomorphic to polymorphic populations.* The transition from domination of individuals with $q = 1$ to domination individuals with intermede value of q, s_0 (solid blue line, Eq. (S1)), decreases and approaches zero as $\alpha \to \infty$. However, the transition from monomorphic to polymorphic populations always appears: s_1 (solid red line) never approaches zero but rather approaches infinity when $\alpha \to 1$ and when $\alpha \to \infty$. Note that $\hat{s}_1$ (dashed red line, Eq. (S6)) well-approximates s_1 for large values of α. Parameters: $F(q) = -q$.
